# Thermotherapy for knee osteoarthritis: A protocol for systematic review

**DOI:** 10.1097/MD.0000000000025873

**Published:** 2021-05-14

**Authors:** Cimin Shen, Na Li, Bin Chen, Jinzuan Wu, Zhining Wu, Dangyun Hua, Lu Wang, Dangdang Chen, Zhuoyi Shao, Changjie Ren, Jinsen Xu

**Affiliations:** a Department of Acupuncture and Moxibustion, Fenghua Hospital of Traditional Chinese Medicine, Ningbo, Zhejiang Province; b Research Department of Meridian, Fujian Institute of Traditional Chinese Medicine, Fuzhou, China.

**Keywords:** knee osteoarthritis, protocol, systematic review, thermotherapy

## Abstract

**Background:**

Osteoarthritis of the knee is one of the leading causes of pain and disability among adults. Thermotherapy has been widely used to treat knee osteoarthritis. But its efficiency has not been scientifically and methodically evaluated. The aim of this study is to assess the benefits of thermotherapy for people with osteoarthritis of the knee, in terms of pain, stiffness, and physical dysfunction.

**Methods:**

Eight databases will be searched from their inception to September 2020. They are as follows: PubMed, Embase, Cochrane Library, ClinicalTrials.gov, China Knowledge Resource Integrated Database (CNKI), Weipu Database for Chinese Technical Periodicals (VIP), Chinese Biomedical Literature Database (CBM), and Wanfang Database. Two researchers will independently select studies, collect data, and assess the methodology quality by the Cochrane risk of bias tool.

**Results:**

The systematic review will provide high-quality evidence to assess the benefits and harms of thermotherapy for people with osteoarthritis of the knee, in terms of pain, stiffness, and dysfunction of knee joint, and quality of life, as well as adverse events.

**Conclusion:**

The systematic review will provide evidence to assess the effectiveness and safety of thermotherapy for knee osteoarthritis patients.

**INPLASY registration number:**

INPLASY202140038.

## 1 Introduction

Knee osteoarthritis (KOA) is one of the leading causes of pain and disability among adults.^[1]^ Its prevalence has steeply increased during the last 6 decades^[2]^; approximately 10% of the world's population aged 60 or older have symptomatic osteoarthritis of the knee.^[3]^ Globally, hip and knee osteoarthritis rank among the top 20 contributors to disability of all health conditions.^[4]^ Typical clinical features include pain, swelling, stiffness, and limited range of motion of the knee joint, often accompanied by a substantial decrease in quality of life. There is no cure for OA at present, and so objectives of management of symptoms of OA of the knee are to lessen pain and stiffness, maintain or improve mobility, and minimize disability.^[5–7]^ Treatment options include pharmacologic intervention, exercise therapy, surgery, and hot and/or cold therapy. Different physiotherapy treatments have been shown to help improve clinical symptoms and function of knee OA with fewer adverse effects than medical treatment. Thermotherapy is 1 such noninvasive therapy.

Thermotherapy is the application of heat to the body resulting in increased tissue temperature.^[8,9]^ Techniques for thermotherapy include the application of moxibustion, hot packs, superficial heat, and via diathermy (application of electromagnetic energy). Thermotherapy is used in rehabilitation to reduce pain and stiffness, and to increase mobility.^[10]^ Thermotherapy helps to relax muscles and increase circulation to the affected area, thus reducing pain and stiffness, although there is some concern that this may, in turn, worsen inflammation and edema. Thermotherapy can be self-applied easily by the patient at home (such as the use of heat packs), and may also be combined with other rehabilitation interventions.^[11]^ There is only 1 review paper of thermotherapy in the treatment of knee osteoarthritis nearly 10 years ago. Since only 3 articles with more than 20 years have been included, there have been reports in recent years that different forms of thermotherapy have been widely used in clinical practice. It has been used to treat knee osteoarthritis and has achieved significant results, especially a kind of thermotherapy called moxibustion, which is commonly used in Chinese hospitals.

Therefore, it is necessary to reassess the efficacy and safety of thermotherapy for KOA. In this study, evidence-based medicine will be used to analyze and evaluate clinical randomized controlled trials (RCTs) in patients with KOA.

## 2 Methods

This protocol for this review was developed in accordance with the PRISMA-P guidelines and the Cochrane Handbook. This protocol has been registered on INPLASY (registration number: INPLASY202140038: https://inplasy.com/inplasy-2021-4-0038/). Ethical approval is unnecessary because this is a literature-based study.

### 2.1 Inclusion criteria for study selection

#### 2.1.1 Types of studies

All RCTs of thermotherapy for KOA without publication status restriction or writing language. Non-RCTs, quasi-RCTs, uncontrolled trials, reviews, case-controlled studies, animal trials, and laboratory studies will be excluded.

#### 2.1.2 Types of patients

We will include participants with osteoarthritis of the knee (as defined by the study). There will also be no limitations related to age, sex, disease duration, and disease severity

#### 2.1.3 Types of interventions

Interventions using thermotherapy only were included in this review. Trials that compared thermotherapy with standard treatment and/or placebo were included. thermotherapy with another active therapy vs the same therapy alone will also be investigated. Trials comparing head to head therapies, such as 2 different types of diathermy, were not included in this review.

#### 2.1.4 Types of outcome measures

##### 2.1.4.1 Major outcomes

The primary outcome is Western Ontario and McMaster Universities Osteoarthritis Index. Western Ontario and McMaster Universities Osteoarthritis Index is a self-report questionnaire for OA of the hip or knee, with higher scores indicating more serious pain, poorer physical function, and increased stiffness. It has been widely used as a tool by clinical investigators to assess patients with KOA.

##### 2.1.4.2 Secondary outcomes

Lequesne Index and Medical Outcomes Study Short Form 36 health survey will be accepted as the secondary outcomes.

##### 2.1.4.3 Safety outcomes

The incidence and severity of side effects will be used to evaluate the safety. Any unexpected events occurred will be recorded.

### 2.2 Search methods for the identification of studies

#### 2.2.1 Electronic searches

Eight databases will be searched from their inception to September 2020. They are as follows: PubMed, Embase, Cochrane Library, ClinicalTrials.gov, China Knowledge Resource Integrated Database (CNKI), Weipu Database for Chinese Technical Periodicals (VIP), Chinese Biomedical Literature Database (CBM), and Wanfang Database. There will be no limitation to study publication status or language. The search terms include KOA, gonarthrosis, osteoarthrosis, osteoarthropathy, arthralgia, thermotherapy, diathermy, heat therapy, Moxibustion, and RCTs. The equivalent search words will be used in the Chinese databases. The detailed strategies for searching the PubMed database will be presented in Table 1.

**Table 1:** Search strategy used in PubMed.

Search	Search terms
1	(((((((((knee osteoarthritis) OR osteoarthritides) OR osteoarthrosis) OR osteoarthroses) OR gonarthrosis) OR osteoarthropathy) OR arthralgia) OR degencrative arthritides) OR ddegencrative arthritis) OR osteoarthrosis deformans
2	((((Thermotherapy) OR hyperthermia) OR diathermy) OR heat therapy) OR moxibustion
3	(((random[Text Word] OR randomized[Text Word]) OR control[Text Word]) OR controlment[Text Word]) OR trial[Text Word] AND “humans”[MeSH Terms]
4	#1 AND #2AND #3

#### 2.2.2 Searching other resources

Additionally, the international clinical trials registry platform, dissertation, and gray literature will also be searched to identify systematic reviews related to thermotherapy for KOA. The relevant conference papers, journals will be retrieved manually.

### 2.3 Data collection and analysis

#### 2.3.1 Selection of studies

**Figure 1:**
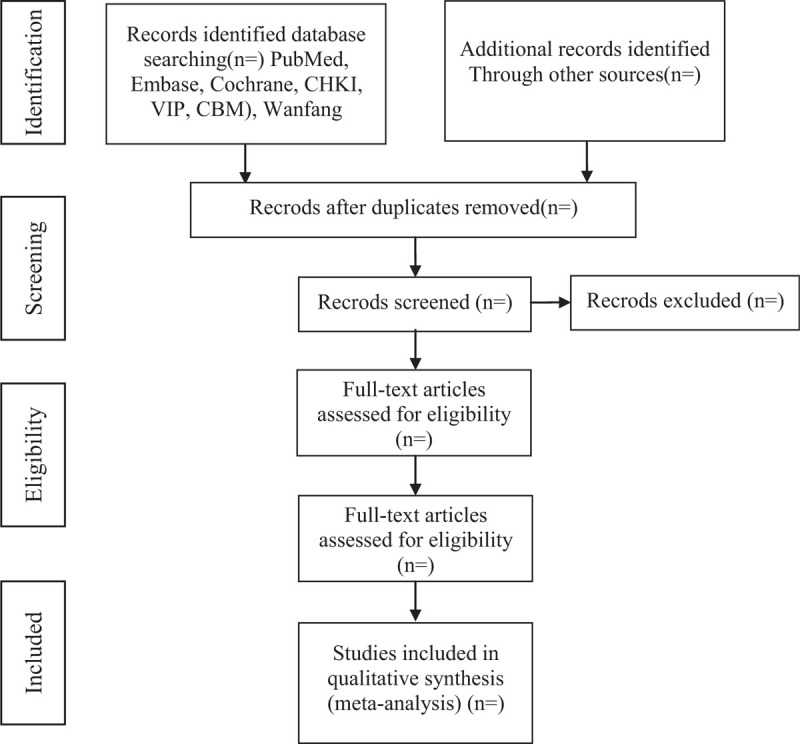
The PRISMA flow diagram of study selection process. PRISMA = Preferred Reporting Items for Systematic Review and Meta-Analysis.

For the first version of the review, 2 authors will screen the search yield and identify any potentially eligible citations. We will retrieve the full text of articles judged as being potentially eligible by at least 1 review author. Two review authors will screen the full-text articles for eligibility and resolve any disagreements by discussion or by consulting a third review author.^[12]^ We will record the selection process in sufficient detail to complete a study selection flow diagram (Fig. 1).

#### 2.3.2 Data extraction and management

Before data extraction, a standard form will be prepared for data collection. Two researchers will independently extract data of the included studies and write on the form. Any disagreement will be solved by consensus. The following data will be extracted: the first author, publication year, participants characteristics, interventions, duration of treatment, follow-up, outcome assessment, research results, adverse events, and other detail information. We will contact the original author for complete information when necessary.

#### 2.3.3 Assessment of risk of bias

Assessment of risk of bias. Two researchers will assess the risk of bias of included studies independently according to the Cochrane collaboration's tool.^[13]^ The tool comprise 7 aspects which are random sequence generation, allocation concealment, the blinding method for patients, researchers and outcomes assessors, incomplete outcome data, and selective reports.^[14]^ Every risk of bias will be classified as low, unclear, and high.

#### 2.3.4 Measures of treatment effect

For continuous data, a mean difference or standardized mean difference with 95% confidence intervals (CIs) will be applied. For dichotomous outcome data, the risk ratio with 95% CIs will be used to evaluate the treatment effect.

#### 2.3.5 Missing data management

If the essential data are not provided, we will try to contact the corresponding author of the articles by email for complete data. If the missing data cannot be obtained, we will analyze the available data.

#### 2.3.6 Assessment of heterogeneity

We will assess clinical and methodological diversity in terms of participants, interventions, outcomes, and study characteristics for the included studies, to determine whether a meta-analysis is appropriate.^[15]^ When meta-analysis is appropriate, we will assess and quantify the possible magnitude of inconsistency (i.e., heterogeneity) across studies, using the *I*^2^ statistic with a guide for interpretation as follows: 0% to 40% might not be important; 30% to 60% may represent moderate heterogeneity; 50% to 90% may represent substantial heterogeneity; and 75% to 100% represents considerable heterogeneity. If we identify cases of considerable heterogeneity (defined as *I*^2^ of 75% or greater), we will explore the data further by comparing the characteristics of individual studies and performing subgroup analyses.

#### 2.3.7 Data synthesis

We plan to pool outcomes from trials with similar characteristics (participants, interventions and common comparators, outcome measures, and timing of outcome measurement) to provide estimates of benefit and harm. We plan to synthesize effect estimates using a random-effects meta-analysis model based on the assumption that clinical diversity is likely to exist, and that different studies are estimating different intervention effects. Where we cannot pool data, we plan to present effect estimates and 95% CIs of each trial in tables, and summarize the results in text.

#### 2.3.8 Sensitivity analysis

Sensitivity analysis. When there are sufficient studies, sensitivity analysis will be performed to assess the robustness of studies according to methodological quality, sample size, and missing data.

#### 2.3.9 Reporting bias

In order to determine whether outcome reporting bias is present, we will check a priori trial protocols against published reports of trial results (i.e., check if all planned outcomes have results reported). We will compare the fixed-effect estimate against the random-effects model to assess the possible presence of small-sample bias in the published literature (i.e., in which the intervention effect is more beneficial in smaller studies).^[16]^ In the presence of small-sample bias, the random-effects estimate of the intervention is more beneficial than the fixed-effect estimate. If we are able to pool more than 10 trials, we will undertake formal statistical tests to investigate funnel plot asymmetry to detect the possibility of publication bias.

#### 2.3.10 Confidence in cumulative evidence

The quality of evidence will be assessed based on the grading of recommendations assessment, development, and evaluation system, include 4 levels: high, moderate, low, or very low.

## 3 Interpreting results and reaching conclusions

We will follow the guidelines in Chapter 12 of the Cochrane Handbook for Systematic Reviews of Interventions for interpreting results, and will be aware of distinguishing a lack of evidence of eHect from a lack of eHect.^[17]^ We will base our conclusions only on findings from the quantitative or narrative synthesis of included studies for this review. We will avoid making recommendations for practice, and our implications for research will suggest priorities for future research and outline what the remaining uncertainties are in the area.

## Author contributions

**Conceptualization:** Na Li, Jinsen Xu.

**Data curation:** Na Li, Bin Chen.

**Formal analysis:** Zhining Wu, Jinsen Xu.

**Funding acquisition:** Jinzuan Wu.

**Investigation:** Dangyun Hua.

**Methodology:** Dangdang Chen, Zhuoyi Shao, Jinsen Xu.

**Project administration:** shen cimin, Na Li.

**Resources:** Na Li.

**Software:** Zhining Wu, Changjie Ren, Jinsen Xu.

**Supervision:** Na Li, Jinsen Xu.

**Validation:** Na Li, Zhining Wu, Lu Wang, Jinsen Xu.

**Visualization:** Na Li, Zhuoyi Shao, Changjie Ren.

**Writing – original draft:** Na Li, Zhining Wu, Zhuoyi Shao.

**Writing – review & editing:** shen cimin.
